# Bioaccumulation, Distribution and Biotransformation of Cylindrospermopsin in Potato (*Solanum tuberosum*) After Exposure by Surface or Sprinkler Irrigation

**DOI:** 10.3390/toxins17060301

**Published:** 2025-06-12

**Authors:** Fredy Duque, Ana Isabel Prieto, Antonio Cascajosa-Lira, Luis Carlos Montenegro, Alexandre Campos, Angeles Jos, Ana M. Cameán

**Affiliations:** 1 Environmental Engineering, Universidad de Cundinamarca Extensión Facatativá, Facatativá 253052, Colombia; faduque@ucundinamarca.edu.co; 2 Area of Toxicology, Faculty of Pharmacy, Universidad de Sevilla, C/Profesor García González 2, 41012 Sevilla, Spain; aclira@us.es (A.C.-L.); angelesjos@us.es (A.J.); camean@us.es (A.M.C.); 3 Algae Culture Laboratory (LAUN), Universidad Nacional de Colombia, Bogotá 110001, DC, Colombia; lcmontenegror@unal.edu.co; 4 Interdisciplinary Centre of Marine and Environmental Research (CIIMAR), University of Porto, Terminal de Cruzeiros do Porto de Leixões, 4450-159 Matosinhos, Portugal

**Keywords:** bioaccumulation, biotransformation, cyanotoxin, soil irrigation, sprinkler irrigation, UHPLC-MS/MS, ICP-OES

## Abstract

Cylindrospermopsin is an emerging cyanotoxin that can lead to phytotoxicity through different mechanisms. The presence of CYN in irrigation waters is of concern due to potential accumulation in plants, increasing the risk of human exposure by the consumption of vegetables. In this case, it is proposed to evaluate the effects of CYN on a crop considered staple food in Colombia, such as *Solanum tuberosum*, group Phureja var Criolla Colombia, known as “yellow potato”. This work evaluates for the first time the effects of CYN in potato plants exposed to this toxin using two different irrigation systems, surface and sprinkler irrigation. The parameters evaluated were CYN bioaccumulation and biotransformation in different parts of the potato plants irrigated with water containing CYN at environmentally relevant concentrations (84.65, 33.80, 3.05 and 3.05 µg/L after first, second, and third to fourth applications, respectively) and changes in nutritional mineral content in tubers. For this purpose, the concentrations of CYN and its potential metabolites in leaves, stem, roots, and tubbers of the plants exposed to the toxin were determined by Ultra-high Performance Liquid Chromatography–MS/MS (UHPLC-MS/MS). Mineral content was determined by Inductively Coupled Plasma Optical Emission Spectroscopy (ICP-OES). CYN bioaccumulation was detected only in aerial parts of plants with sprinkler irrigation. A total of 57 CYN metabolites were found, and the main differences obtained in CYN biotransformation are linked to tissues and exposure conditions. There are significant differences in levels of Ca, K, Mg, Na, P, Cu, Fe, Mn, and Zn in tubers depending on CYN treatment, with higher contents after surface irrigation, and lower content with sprinkler application. These results demonstrate that the exposure conditions are an important factor for the potential presence and effects of CYN in potato plants.

## 1. Introduction

Cyanobacteria, also known as blue-green algae, are prokaryotic organisms that thrive in nutrient-rich (eutrophic) freshwater environments like lakes. Their ability to produce toxins called cyanotoxins is of significant concern, as these toxins pose a threat to both aquatic life and humans [1,2]. Cyanotoxins can be classified in two ways, either according to their chemical structure or their toxic effects [3]. Among them, Cylindrospermopsin (CYN) is a bioactive compound consisting of a sulphate ester with a tricyclic guanidine moiety attached to a hydroxymethyluracil molecule (C_15_H_21_N_5_O_7_S; molecular weight 415.43 g/mol) [4,5].

CYN has been reported to be toxic to humans, animals, and plants [6,7,8,9]. The toxin can also cause oxidative damage [8]. CYN, which inhibits protein and glutathione synthesis [10,11], was originally characterized as hepatotoxic but it has been also identified as potentially genotoxic, carcinogenic, dermatotoxic, and immunotoxic, among others [9,12,13]. Specifically, some studies reported that CYN is pro-genotoxic and needs to be metabolically activated by cytochrome P-450 enzymes to become genotoxic [14].

CYN has emerged in recent decades as a cyanotoxin of increasing concern [9,15]. This is due in part to the high stability of CYN under different pH and temperatures, persisting in the environment for long periods, even after bloom dissipation [16]. Therefore, the widespread presence of this toxin and its high water solubility (due to high polarity) pose a significant health hazard, particularly when these water sources are utilized for human consumption, agricultural irrigation, recreational purposes, and animal consumption [5].

Even though cyanotoxins pose a serious hazard to animals and humans on their own, the exposition pathways will determine the effects in health. The main routes of exposure are oral, through the consumption of contaminated water and food, and dermal. Concerning contaminated food, one of the main sources is the cyanotoxins uptake and bioaccumulation by crops irrigated with toxin-contaminated waters [17].

The accumulation of cyanotoxins in plants can lead to phytotoxicity through various mechanisms. These include increased oxidative stress, inhibition of seed germination, hindered seedling growth and development, suppression of regulatory enzyme activities, damage to plant tissues, and overall crops loss [18]. In general, these main phytotoxic effects of cyanotoxin in plants, including effects on nitrogen and mineral uptake, differ for different cyanotoxins, the exposure concentration, and the genotype of the exposed plant species [19]. In edible plants specifically, Prieto et al. (2011) indicated that CYN can induce oxidative stress in rice (*Oryza sativa*) [20], and M-Hamvas et al. (2010) demonstrated that it altered the growth, development and peroxidase enzyme activity of white mustard seedlings (*Sinapis alba*) [21]. Moreover, CYN has been linked to chromatin alterations, chromosomal aberrations, and mitotic retardation in *Vicia faba* cells, according to the results obtained by Garda et al. (2015) [22]. Interestingly, Llana-Ruiz-Cabello et al. [23] have shown that photosynthetic capacity in lettuce (*Lactuca sativa*) and spinach (*Spinacia oleracea*) remains unaffected when exposed to CYN concentrations.

Moreover, the uptake of toxins by plants can cause both morphological and physiological alterations, resulting in reduced productivity. To our knowledge, the effects of CYN on potato plants have been not yet studied. Only one study on *S. tuberosum* plants exposed to microcystin-LR (MC-LR) cyanotoxin showed some adverse developmental effects such as 25 ± 50% tissue necrosis in potato shoot cultures and a substantial reduction in biomass and chlorophyll content [24]. Additionally, it can significantly affect their nutritional value and lead to the bioaccumulation of toxins. Thus, CYN has been reported to affect the nutritional content of lettuce (*L. sativa*) [25] and carrots (*Daucus carota*) [26], being the first study on a vegetable whose root is edible. In relation to the bioaccumulation of CYN in crops, there are reports of accumulation in *Brassica oleracea* var. sabellica, *Brassica juncea*, *S. alba* [27], *L. sativa* [23,28], in *S. oleracea* [23] and *Eruca sativa* [28]. All the reports concluded that bioaccumulation depends on the exposure concentration, exposure time, and on plant genotype. Therefore, CYN is present and can bioaccumulate in plants. On the contrary, no biomagnification processes have been described, probably due to the hydrophilic nature of the toxin and the presence of detoxification mechanisms.

Keeping in mind the human health risks associated with the bioaccumulation of cyanotoxins [29,30], attention should be paid to agricultural crops under field conditions [31,32], specifically in agri-food systems that present important opportunities for the rural population, in terms of food security, poverty alleviation, and improved health status [33]. Potato is now the world’s third most important food crop for human consumption, after wheat and rice [34]. Devaux et al. [35] reported that world potato production is shifting towards developing countries, with a strong increase in production and harvesting areas in Asia, and on lower scale, in East Africa and Latin America and the Caribbean, to such an extent that potato production in developing countries exceeded that of developed countries for the first time in 2005 [34]. This is the case of Colombia, with a genetic breeding program for native potatoes of the Phureja group “the yellow potato” [36], which has made it possible both to increase the production of established varieties and to provide farmers with new varieties [37]. In addition, potato is considered a superfood crop due to the presence of vitamins and minerals, phytochemicals, protein with high nutritive value, dietary fiber and resistant starch content and low-calorie content, and absence of fat, cholesterol, sodium and gluten [38].

The potential bioaccumulation of cyanotoxins in crops irrigated with contaminated water claims for assessing human risks derived from their consumption [39]. Therefore, due to the recurrent identification of CYN as a potential contaminant in irrigation systems, the study of the uptake and accumulation in plants is indispensable [19]. In this sense, diverse methodologies for the extraction and determination of CYN in vegetable matrices have been developed, based on a previous solid phase extraction of the toxin and detection and quantification by Ultra Pressure Liquid Chromatography–Mass Spectrometry in tandem (UPLC-MS/MS) [40].

Considering all these facts, this work is the first one focused on the evaluation of the presence and potential bioaccumulation of CYN in potato plants (*S. tuberosum*) exposed by two different irrigation systems, by surface and sprinkler irrigation. Thus, the objectives of the present work were 1—to determine the potential bioaccumulation of CYN in the different parts of the potato plants irrigated with water containing CYN at environmentally relevant concentrations (3.05–84.65 µg/L); 2—to know its biotransformation, as well as 3—the potential changes in mineral content of the tubers at the exposure conditions assayed. For this purpose, the concentrations of CYN and its potential metabolites in leaves, stem, roots, and tubers of the plants exposed to the toxin were measured by Ultra-High Performance Liquid Chromatography–MS/MS (UHPLC-MS/MS). Moreover, the mineral content was determined (Ca, K, Mg, Na, P, Cu, Fe, Mn and Zn) by Inductively Coupled Plasma Optical Emission Spectroscopy (ICP-OES).

## 2. Results

### 2.1. Bioaccumulation of CYN in Plant Tissues

After 30 days of sprinkler irrigation with CYN-contaminated water, no CYN was detected in roots and tubers. By contrast, CYN was detected in leaves and stems (Table 1). The concentrations of CYN found ranged between 0.07 and 0.16 µg/g d.w (equivalent to 3.07–8.28 µg/L) in leaves, and between 0.04 and 0.12 µg/g d.w. (equivalent to 1.98–5.75 µg/L) in stems. In contrast, no CYN was detected in any tissue of plants treated by surface irrigation (Table 1). Control samples were also measured, and CYN was not detected.

**Figure 1:**
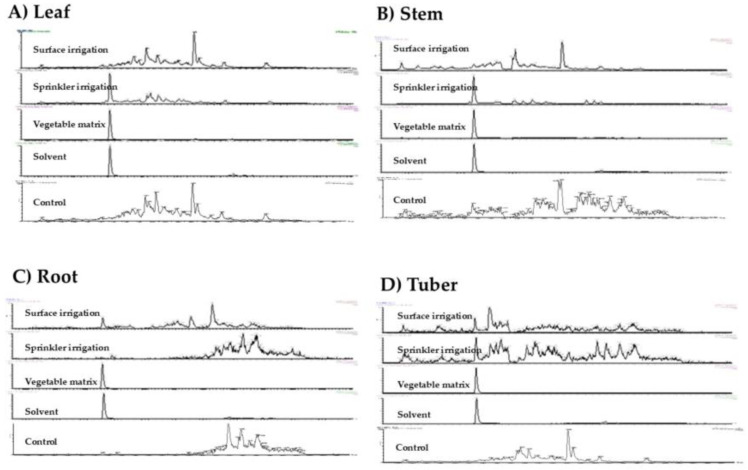
UPLC-MS/MS chromatograms displaying the retention time and peak areas of CYN in various plant organs of *S. tuberosum* exposed to CYN through different methods. The chromatograms represent (**A**) leaf, (**B**) stem, (**C**) root, and (**D**) tuber. The study includes samples from surface irrigation, sprinkler irrigation, CYN standard in *S. tuberosum* matrix, CYN standard in solvent samples, and control.

Figure 1 shows the UPLC-MS/MS chromatograms obtained in the different parts of the potato plants exposed to CYN by the two irrigation systems employed.

### 2.2. Biotransformation of CYN in the Plant Tissues

**Figure 2:**
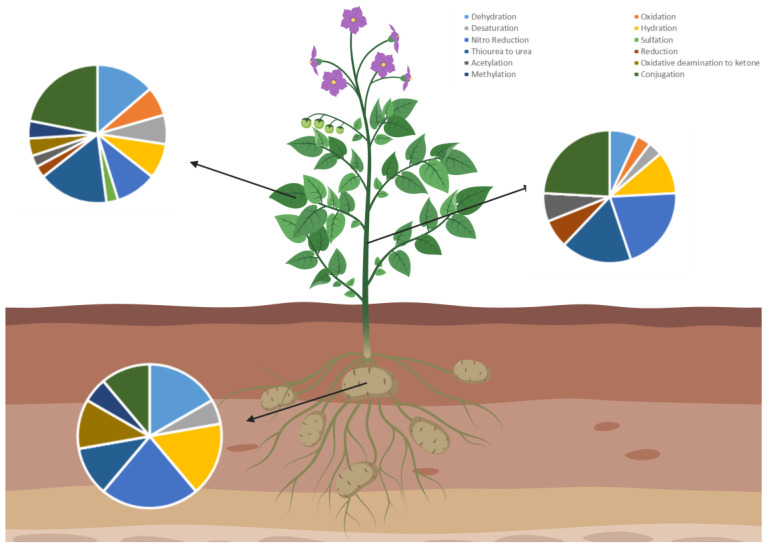
Summary of the main CYN transformation products detected in the different parts of *S. tuberosum*. Created with Biorender.com.

The extracts of the different tissues of the plants were analyzed by UHPLC-MS/MS and submitted to Compound Discoverer™ 3.2 to identified possible CYN metabolites. No CYN metabolites were detected in the control samples and in roots of the plants exposed to CYN using both irrigation systems. However, a total of 57 CYN metabolites were detected and identified in leaves (*n* = 35), stems (*n* = 13), and tubers *(n* = 9) (Table 2). The metabolites resulting from phase I reactions include dehydration, desaturation, hydration, nitro reduction, oxidative deamination to alcohol, oxidative deamination to ketone, oxidation, reduction, and transformation of thiourea to urea. Phase II conjugation reactions produce metabolites through acetylation, methylation, sulfation, and conjugation with arginine, cysteine, glucoside, glucuronide, glutamine, glycine, ornithine, palmitoyl, stearyl, and taurine (Figure 2). Although similar values were found with both irrigation methods, there are differences between parts of the plant. Thus, after sprinkler irrigation, the plant organs with higher number of different CYN metabolites (qualitative analysis) are leaves > stems > tubers while in surface irrigation the order was leaves > tubers > stems.

### 2.3. Effects of CYN on Tubers of Solanum Tuberosum Mineral Content

In order to disclose the physiological and nutritional quality effects caused by the exposure to the toxin, the content of macronutrients (Ca, K, Mg, Na P) and micronutrients (Cu, Fe, Mn, and Zn) was determined in tubers of potatoes exposed to CYN by both irrigation systems. The interval of variation as well as mean values (±standard deviation, SD) of these mineral contents are shown in Table 3.

Some other data found in the scientific literature were included in this table, for comparison.

**Figure 3:**
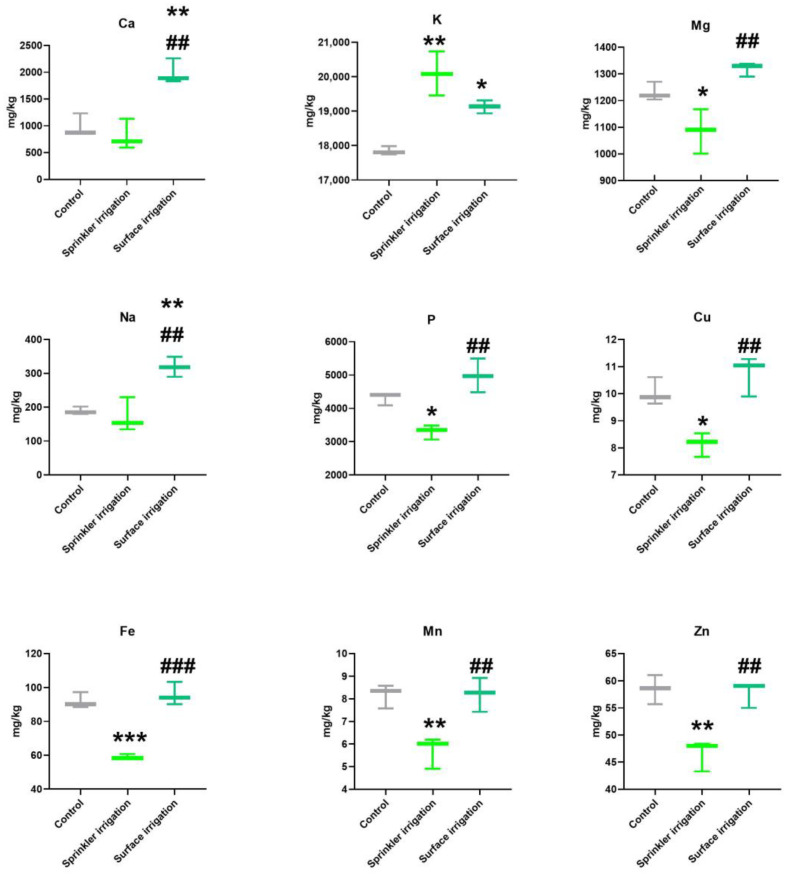
Box–whisker plots of macronutrients (Ca, K, Mg, Na and P) and micronutrients (Cu, Fe, Mn, Zn) content of tubers from potato plants exposed to CYN using different irrigation systems: surface or sprinkler. Values are mean ± standard deviation (SD). (* *p* < 0.05, ** *p* < 0.01, *** *p* < 0.001). *—Statistical differences compared with the control group. (## *p* < 0.01, ### *p* < 0.001). #—Statistical differences compared with surface irrigation group.

Globally, the contents of macronutrients in plants exposed to CYN by surface irrigation were increased in comparison to their respective controls (Ca, K, Na, P), while the sprinkler irrigation of leaves with the toxin only elevated the levels of K and significantly decreased the contents of Mg and P (Figure 3).

Concerning the impact of CYN on micronutrients content (Cu, Fe, Mn, Zn) in potato tubers, in general, significant declines in the content of the four minerals in tubers exposed by sprinkler irrigation were observed. Control plants and plants after sprinkler irrigation were exposed under comparable experimental conditions (see Section 5.3), and consequently, the mineral content decreases could be explained by the effects of CYN exposure. In contrast, after surface irrigation with CYN, no significant differences in the mineral content of Cu, Fe, Mn and Zn were found in comparison with control tubers. There are significant differences in levels of Cu, Fe, Mn and Zn in tubers depending on the CYN exposure system, with higher contents after surface irrigation and lower contents by sprinkler application of the toxin.

## 3. Discussion

### 3.1. Bioaccumulation of CYN in Potato Plants

The uptake and bioaccumulation of CYN in potato plants (*S. tuberosum*) grown in natural soil in greenhouse and irrigated with CYN contaminated water at significant environmental concentrations, simulating crop field conditions, was evaluated for the first time in the present work. Two irrigation systems were evaluated: sprinkler and surface irrigation. Both control and exposed plants were sampled at the end of the treatment. Given the novelty of this experiment, higher concentrations of CYN were chosen in the first applications, and then it was decided to lower them to avoid important or unexpected deterioration of the exposed plants. The results indicated that CYN was detected in leaves and stems after sprinkler irrigation with CYN contaminated water but not with surface irrigation. Additionally, it should be noted that CYN was not detected in roots or tubers in any of the experimental procedures.

The effects of *C. raciborskii* extracts on the germination and growth of several plant species, including *S. lycopersicum*, have been previously studied [48], although CYN bioaccumulation data in this species were not reported. CYN accumulation has been demonstrated in other crops, in leaves and roots [19]. Globally, CYN uptake and accumulation in plants are governed by selective factors such as the CYN concentration, the type of the plant part exposed to CYN, the exposure time, the plant species, etc. [19,20,23]. The importance of these factors is demonstrated in different studies, which indicated variable results in relation to CYN bioaccumulation in several parts of the plants. Thus, the presence of CYN in aerial parts of potato plants after sprinkler application agrees with the results reported in lettuce and spinach reported by Cordeiro-Araujo et al. [28]. The authors applied 3, 5 and 10 µg/L CYN directly to plants for 7 days and found that leaves of lettuce and spinach accumulated CYN (not data on roots were reported). The authors indicated that at the higher exposure concentrations the bioaccumulation of CYN was lower. In addition, there are reports of CYN accumulation in different plant parts in hydroponic assays. Thus, Kittler et al. [27], in a soil-free aeroponic cultivation system, showed significant uptake in *B. oleracea, B. juncea* and *S.alba* under several experimental conditions, with CYN levels ranging from to 10% to 21% in the leaves compared to the CYN concentration applied to the roots. CYN bioaccumulation was also different with CYN transfer to the stem in several edible plants, such as *Phaseolus vulgaris*, *Pisum sativum* and *L. sativa* [48]. The CYN bioconcentration was plant and concentration dependent in the roots and stem of these plants, and the species *L. sativa* and *P. sativum* had the lowest root CYN concentration, which might indicate rapid translocation to the stem and/or more efficient detoxication of CYN in the roots. In addition, Prieto et al. [20] detected CYN in both leaves and roots of *O. sativa* exposed for 48 h to *C. ovalisporum* cell extracts containing 2.5 mg/L CYN, and lower concentrations in the leaves were found in comparison to the roots.

On the other hand, when spinach and lettuce were exposed to CYN and Microcystins (MCs) mixtures (at 10 µg/L and 50 µg/L, respectively) for 21 days by sprinkler irrigation under hydroponic conditions, differential accumulation of CYN and MCs was reported, and only CYN was translocated from the roots to the leaves of exposed plants [23]. In this case, CYN accumulation in lettuce roots was higher than in the leaves; conversely in spinach, the toxin was accumulated in leaves in higher quantities. In sprinkler irrigation, the xenobiotic uptake for plants depends on the following variables: (1) direct contact with the foliar surface, this has been already reported for heavy metals, organic compounds and pesticides [49,50], (2) properties of the leaf cuticle, and environmental conditions at the time of application [51], and (3) chemical properties of the xenobiotic per se [52], highlighting its hydrophilic character. CYN must be transported from the leaf surface through the cuticle to reach internal plant tissues. The vascular system is the relevant route of CYN transport within the plants, which leads to the accumulation of CYN in other plant organs in addition to roots [23]. With sprinkler irrigation, the detection of CYN in stems could indicate that the molecule could be translocated from leaves to stems via the phloem. Moreover, it may also explain that the substance had a low cuticular sorption since the values of CYN bioaccumulated were much lower than the concentrations applied and low mobility since it was not detected in roots or tubers [51]. These hypotheses could explain the CYN bioaccumulation in aerial parts of potato plants after CYN exposure by sprinkler irrigation. Differences in CYN accumulation found depending on the part of the plant could be also explained in part by the direction of application of the contaminated water. This supports that the toxin was only detected in leaves and stems of sprinkler-irrigated plants and not detected in any of the surface-irrigated plant parts.

According to our results obtained in plants after surface irrigation, Pereira et al. [53] applied CYN crude extract at three concentration levels, 0.1, 0.5 and 1 µg/L, directly in seedlings of parsley and coriander grown in non-sterile soil and found that CYN was not accumulated in the edible leaves and stems. In surface irrigation, cyanotoxins could be transferred from irrigation water to soil [54], and subsequently they may be transformed or degraded by soil microbiota [15]. In addition, soil content may influence the disposition of toxins by the plant. In the present work, the content of the soil employed showed a high organic carbon content of 11.3%, so it is possible that it could be involved in the adsorption and degradation of CYN. As it has been exposed above, the polarity of CYN is a factor to be considered [9,55]. CYN is a hydrophilic zwitterion that carries both a positive and a negative charge at pH 6–8.5, with a high polarity and a strong tendency to remain in aqueous solution. Zhang et al. [55] indicated that the high polarity of CYN together with the electrostatic repulsions between negatively charged clay/organic matter surfaces and the anionic sulfur group in CYN, causes a low sorption of CYN by the soil, and it has been classified as very low sorptivity in soils. Chen et al. [56], in a batch experiment in non-sterile soil, reported that the adsorption mechanism of MCs in soil is due to chemical binding with the metal ions on the surface of particles and increased toxin degradation was verified in soils with high contents of clay and organic matter. However, adsorption studies of MC-LR and nodularin (NOD) revealed that soils with high clay (16.9%) and organic carbon (2.9%) contents had higher toxin adsorption coefficients [57].

Concerning MCs, the degradation of this toxin is influenced by environmental factors such as light, water composition, presence of organic matter, microbial activity, and physicochemical properties of the soil [58,59]. However, studies focused on the fate of CYN in the soil and their effects in microbiota of cultivated soils are still scarce [60]. Finally, there is a growing interest about the mobility of CYN in soils, its fate, its potential degradation or bioaccumulation [61], which has led to consider the persistence of this toxin in agricultural soils [62] and it is a subject that requires further research.

In the *Solanum* genus, a mean concentration of 11.55 µg/mL of MC-LR was detected in the extracts of *S. tuberosum* plants exposed to the toxin through the growth medium for 3 days (approximately 96% of the amount of toxin). This amount gradually decreased to 32%, 23% and 18% after 6, 12 and 18 days of exposure, respectively [24]. In addition, Corbel et al. [63] observed MCs uptake in *S. lycopersicum* grown in silty soil, with MC-LR concentrations in tomato leaves ranging from 0.29 to 0.55 μg/kg d.w. when exposed to cyanobacterial extracts containing 20 to 100 μg/L MC-LR. In roots, concentrations ranged from 4.5 to 8.1 μg/kg d.w. across all extract concentrations (5 to 100 μg/L MC-LR). Similarly, Gutiérrez-Praena et al. [17] reported MCs bioaccumulation in *S. lycopersicum* grown hydroponically, with lower concentrations detected in green tomatoes (5.15–5.41 μg/kg f.w.). Higher concentrations were found in mature tomatoes (10.52–10.83 μg/kg f.w.), roots (1635.21 μg/kg f.w.), and leaves (27,673.21 μg/kg f.w.). During the second week of exposure, concentrations in fruits decreased and were undetectable. This decline may be attributed to chemical modification or metabolism of the toxin within plant tissues, or its sequestration through binding biomolecules such as protein phosphatase.

### 3.2. CYN Uptake and Biotransformation in Plant Tissues

A total of 57 CYN metabolites were detected in different parts of *S. tuberosum*: leaves, stem, and tuber by UHPLC–MS/MS, and under both irrigation systems. Toxin exposure conditions led to accumulation of different metabolization products in the organs analyzed, related to phase I and phase II (Table 2). In leaves from both irrigation types, the highest number of CYN metabolites are generated by phase II reactions being the amino acid conjugates the main metabolites identified. Important differences are found in stems. In surface irrigation the highest number of CYN metabolites are generated by phase II reactions and comprise mainly amino acid conjugates. In contrast, in sprinkle irrigation the highest number of CYN metabolites in stems are generated from transformation of thiourea in urea, sulfation and acetylation. Regarding tubers, CYN metabolites generated by nitro reduction reactions (phase I reaction) predominate. In contrast, a reduced number of phase II metabolites of CYN were identified.

In general, in both irrigation systems the presence of methylation was observed. Moreover, in the surface treatment, palmitoyl and stearyl conjugation was additionally detected. The metabolites found in the present study are similar to those found in the extract of *C. ovalisporum* by Hinojosa et al. [64]. These authors reported that the metabolites detected in the cyanobacteria extract are mostly phase I while in the potato plant we found both phase I and phase II metabolites.

Therefore, in the present work the potato plants have high capacity to metabolize CYN, which leads to the need to investigate the toxicity of some of those metabolites. Some authors have shown that the toxicity of CYN is mainly due to the presence of the uracil moiety [65], so those metabolites that have undergone biotransformation on this group are expected to be less toxic than CYN.

### 3.3. Changes in Mineral Content of Tubers After CYN Exposure

The mineral composition of potato tubers is primarily determined by the availability of mineral elements in the soil, which depends on local geology and agronomic practices, such as conventional, integrated, or organic production methods [45]. Additionally, the metal content in potato crops is influenced by several factors, including soil type and structure, soil pH, redox potential, microbial activity, organic matter and water content, climatic conditions, and crop types and variety choices [66,67]. Understanding the quantitative presence of minerals in potato tubers is important not only for assessing their authenticity and geographical origin but also for identifying potential changes in the distribution of these nutrients when tubers are exposed to contaminants, stress conditions, or toxins.

Concerning the contents of macro and micronutrients in plants exposed to CYN by both irrigation systems, the values obtained in all groups assayed are considered higher than previous reported in this variety of *S. tuberosum* Phureja Group tuber cultivated in Colombia [41] and in Andean potato cultivars [42,43,44] (Table 3). This could be explained by several factors, as mentioned above, including soil type (in this case, with high content in Fe, P, K, Na) and structure, soil pH, redox potential, the activity of microbes, organic matter and water content, climatic conditions, etc. These higher values of minerals in comparison to other commercial potato cultivars, indicate that *S. tuberosum* group Phureja would be a good candidate to select the best for production by communities in Colombia and Andean area. In the present work, there are significant differences in levels of Ca, K, Mg, Na, P, Cu, Fe, Mn, and Zn in tubers depending on the type of CYN exposure; consequently, the exposure conditions (surface irrigation or sprinkler application) are an important factor for the potential presence and effects of CYN in potatoes.

In this study, the presence of CYN increased the calcium (Ca) content in potato tubers when plants were exposed to the toxin through surface irrigation compared to the control group. Calcium plays a crucial role in improving membrane stability and enhancing the resistance of potatoes to environmental stresses such as heat, microbial, and nematode infections [68,69]. The levels of sodium (Na) in potato tubers followed a similar trend to calcium, with increased content observed after exposure to CYN via surface irrigation. Conversely, magnesium (Mg) and phosphorus (P) levels in tubers decreased when CYN was applied to leaves through sprinkling but increased when the toxin was applied via surface irrigation, compared to control tubers. Magnesium is essential as a component of the chlorophyll molecule and plays critical roles in enzyme activation involved in respiration, photosynthesis, and the synthesis of DNA and RNA [23]. Phosphorus is vital for plant growth and reproduction, and is essential for ATP, DNA, and other fundamental biological processes. Adequate levels of phosphorus are necessary for photosynthesis, nitrogen fixation, fruiting, seed production, flowering, and overall plant maturity [70].

Potassium (K) is crucial for the growth and development of plants, contributing to the vigor and efficiency of potato plants, and is well-known for its impact on the storability and quality of potato tubers [43]. K concentrations were higher in tubers exposed to CYN by both irrigation systems, particularly following sprinkler application on leaves. The application of CYN may enhance the mobility of potassium, facilitating its transport from surface layers to the interior of the tuber. In contrast, for magnesium and phosphorus, the opposite effect was observed. The physiological response and consequently the changes in roots mineral content may vary according to the plant species [23,25]. Thus, in agreement with our results, significant increases in K and a significant decrease in Mg content in roots of lettuces were found after CYN or mixtures CYN/MCs exposure, being the effects more pronounced depending of the concentration assayed [23]. Lahrouni et al. [71] proposed that these differences in minerals might result from changes of plant membrane permeability caused by cyanotoxins; in addition, the antioxidant response to stress promoted by cyanotoxins is usually dependent of the part of the plant, being more pronounced in roots than the leaves of exposed plants. In fact, the uptake of nutrients in some edible plants (spinach, lettuce) could be affected by oxidative stress, cellular damage and changes in membrane permeability caused by the cyanotoxins [25].

In relation to the micronutrients measured, the mean level of trace metals in all the groups of potato tubers was Fe> Zn > Cu ≈ Mn, and this order agrees with previous results obtained in other countries [67]. In general, potato tubers are a good source of Fe and Zn, and this order agrees with the values obtained by Peña et al. [41] for potato tubers (*S. tuberosum*) group Phureja. Similarly, in native potato tubers cultivated in Andean locations in Peru the levels of Fe and Zn were the highest among micronutrients analyzed, following the decreasing order: Fe > Zn with respect to these elements [44].

In the current work, Fe contents were similar or higher than the previous values reported in several genotypes of the group Phureja [42,43]. In a similar way, the Zn, Cu and Mn contents in all the groups assayed were similar or greater than those reported for this Colombian tuber (group Phureja) [41,42,43]. In general, the changes of these micronutrient contents follow the same trend: decreased after exposure to CYN applied by sprinkler application on leaves, while surface irrigation maintains the levels compared to the control groups. This pattern was parallel to the effects of CYN on Mg and P contents. These micronutrients play key roles in the physiology, growth, and they are important cofactors in diverse processes (photosynthesis, antioxidant status, etc.) of the plants; thus, Fe and Cu are involved in the synthesis of redox reactions; Mn in part of the structure of some photosynthetic proteins and Zn is essential for the synthesis of chlorophyll and for metalloenzymes [25,26].

Globally, considering mineral concentrations, potatoes appear to be less susceptible to CYN toxicity compared to other root tubers like carrots, as observed in a study where carrots were exposed to different concentrations of a *C. ovalisporum* extract containing CYN (10 or 50 µg CYN/L). A general decrease in Ca, Mg, Na, Mn, Fe, Zn, Mo, and P contents in fully developed roots, particularly at higher CYN concentrations [26] was observed. Only K and Cu levels increased, and the latter was observed only at the lower CYN concentration tested (10 µg CYN/L). The effects of CYN on mineral content have also been demonstrated in other edible vegetables such as spinach and lettuce [23,25]. In the first study, lettuce plants (*L. sativa* L.) exposed to environmentally relevant concentrations of CYN (1, 10, and 100 µg/L) over 5 to 10 days generally showed increased mineral content in leaves (Mn, Fe, Zn, Cu, Mo) with CYN exposure, indicating potential time and concentration-dependent effects. The authors suggested that CYN-exposed lettuce plants retained higher mineral content, especially macronutrients, possibly indicating tolerance to the toxin [25]. In the second study, when lettuce and spinach were grown hydroponically for 21 days and exposed to CYN (10 and 50 µg CYN/L), significant changes in micronutrients were more pronounced in roots than in leaves of both vegetables, resulting in decreased Mn and Cu content in roots. CYN exposure reduced K levels in spinach leaves, while significant increases in K and decreases in Ca and Mg contents were observed in lettuce roots after exposure [23]. These findings are consistent with observations from the present study on potato plants exposed to CYN via leaf sprinkler, showing contrasting effects compared to surface irrigation. These discrepancies may be attributed to various factors including plant species, duration and exposure concentrations, mechanisms of enzymatic and non-enzymatic defense systems, and differences in growth systems.

## 4. Conclusions

This is the first investigation of CYN accumulation in potato plants (*S. tuberosum* Phureja Group var Criolla Colombia) exposed to CYN contaminated water by sprinkle or surface irrigation at significant environmental concentrations. The results show that bioaccumulation is directly related to the irrigation method, and CYN was only detected in aerial parts after sprinkle irrigation. A total of 57 metabolites were detected by UHPLC-MS/MS after CYN exposure, and the main differences obtained in CYN biotransformation are linked to tissues and exposure conditions, with higher contents after surface irrigation, whereas lower results were obtained when the exposure was by sprinkler application of the toxin. Further studies are needed to understand the effects of this toxin in the production and quality of potatoes grown with contaminated water.

## 5. Materials and Methods

### 5.1. Cyanobacterial Culture

CYN crude extract was obtained from a *Chrysosporum ovalisporum* (LEGE X-001) (*Umezakia ovalisporum)* [72] CYN-producing (CYN+) strain. The strain LEGE X-001 is maintained in the Algae Cultivation Laboratory (LAUN) of the National University of Colombia. The cultures were grown in bottles of 250 mL and were scaling for biomass production in bottles of 500 mL, 1000 mL, and 5000 mL. In all cases, the experiment was developed in Z8 liquid medium [73] in a closed photobioreactor with temperature 25 °C +/− 2 °C, and 12:12 light/dark photoperiod and agitation for air.

### 5.2. Cylindrospermopsin Extraction and Quantification from Crude Extract

The CYN crude extract from the cyanobacterial culture was obtained to simulate the possible conditions observed in the environment. The Z8 medium with *C. ovalisporum* strain was passed on fiberglass filters (40 mm diameter, 1.5 µm Whatman 934 AH, Maidstone, UK) with the aid of a vacuum pump system and was recovered in an Erlenmeyer bottle (500 mL) (extracellular fraction). The filtered content (intracellular fraction) was macerated with liquid nitrogen in a mortar, added to the Erlenmeyer bottle where the liquid was previously collected and stirred until total liquefaction of the sample. Then, CYN total concentration (intra an extra cellular fractions) was measured by UPLC-MS/MS [74]. CYN crude extract was stored in glass bottles with GL45 screw caps and maintained at 4 °C.

### 5.3. Plant Material and Exposure to Cylindrospermopsin

Potato plants from the *S. tuberosum* Phureja Group [75] were cultivated in a greenhouse in the Department of Biology of the National University of Colombia in Bogotá city (Cundinamarca, Colombia; altitude 2630 m.a.s.l., latitude 4°35′56″ N and longitude 74°04′51″ W) from July to October of 2023. The seeds are *S. tuberosum* Phureja Group var Criolla Colombia, a variety from Fedepapa.

The crop was performed in semi-controlled conditions, in an air temperature range between 17 and 20 °C with a light–dark cycle of 12:12 h (natural light) and a general average air relative humidity of 55%, which allowed the plants to grow, to flower and to develop tubers. The tubers were planted in plastic bags (60 cm, 48 cm, 0.35 mm thick) that contained organic soil and quarzitic sand at a 3:1 ratio. The substrate was a soil with a fine texture, mildly acidic (pH 5.22) and with a mineral composition characterized by iron (57.3%), phosphorus and potassium (16.9–0.37%), sodium (0.13%), calcium magnesium ratio (5.55–1.01%), organic matter (NT 1.10%) and an effective cation exchange capacity (7.39).

One tuber was sown per bag, maintained at the maximum soil capacity, with optimum nutrients and health as described by Ñústez-López and Rodríguez-Molano [76]. Crops were fertilized with a soil application of 1 g per plant (15:15:15, N:P:K, Forza, Fercon SA, Acopi-Yumbo, Colombia) after 30 days, a foliar application 1 mL/L (10:30:10, N:P:K, Fertitec MK, Tecnoquímicas SA, Cali, Colombia) after 65 days and hilling was carried out after 60 days. Two weeding manually operations were carried out, one after 30 days and second after 60 days (while the hilling is being carried out).

Plants were exposed to the CYN crude extract within the last month, for four weeks and CYN concentration for every application was 84.65 µg/L (1st application), 33.80 µg/L (2nd application), and 3.05 µg/L (3rd–4th applications). Irrigation was applied three times per week with running water at field capacity during the experiment, with similar volume of clean water to each plant (Table 4).

There were 3 trays, with 9 plants per tray for a total of 27 plants (9 plants for CYN surface irrigation, 9 plants for sprinkler irrigation and 9 plants for the control group). In the first tray, CYN exposition was directly in soil near to the stems. This surface irrigation was a controlled and localized free-flush with a volume of 100 mL of CYN-contaminated water per pot. Water with CYN crude extract was applied directly onto the substrate around the main stem with a hose. This type of irrigation was intended to ensure that roots and tubers were exposed to the contaminated water [77]. This application seeks to simulate the way in which hose irrigation is carried out in small crops [78] such as the egg yolk potato in Colombia [79,80]. In the second tray, CYN exposition was in foliar sprinkling. Treatment 3 was the control group, in which the plants were continuously well-watered (tap water) at 100% of the maximum soil capacity. For all the treatments, the water capacity was controlled daily using a WET-2 Sensor/HH2 Moisture Meter (Delta-T-devices, Cambridge, UK), and each bag was watered according to the sensor readings.

After 30 days of CYN exposure, both control and treated plants were harvested, and leaves, stems, roots, and tubers separated in plastic bags. The different parts of the plants were stored at −80 °C to determine CYN concentrations in the control group and both experimental groups. Biotransformation products were also detected. In addition, the mineral content of tubers was also analyzed.

### 5.4. Cylindrospermopsin Extraction and Purification from Vegetal Matrix

The quantification of CYN in potato plants was carried out according to Llana-Ruiz-Cabello et al. [23]

### 5.5. Cylindrospermopsin Metabolites Identification in Plant Tissues by UHPLC-MS/MS

The metabolite study followed the data treatment protocol described by Hinojosa et al. [64]. Compound Discoverer™ 3.2 software (Thermo Fisher Scientific, Waltham, MA, USA) was used for the metabolism studies. Mass spectral data were processed to select spectra and align retention times between blanks and samples.

### 5.6. Determination of Mineral Content in Potatoes

Lyophilized tubers, edible part of the potato plants, from all the exposure groups (control, surface and sprinkler irrigation) were taken for the determination of mineral content (*n* = 3). An estimated amount of 0.1 g of each sample was digested in a Single Reaction Chamber microwave system (Ultrawave, Singapore) by Milestone (Milestone Srl., Sorisole (BG), Italy). The only reagent used for digestion was doubly distilled 65% HNO_3_, obtained by running HNO_3_ 65% PA twice in our subboiling acid purification system DST-1000 from Savillex (Savillex, Eden Prairie, MN, USA) [81]. Digestion was performed in two different stages. At the first stage, the sample was let to sit with 3 mL of double-distilled 65% HNO_3_ for 24 h. At the second stage, the remaining 2 mL (up to 5 mL) of HNO_3_ were added to the digestion vessels already containing the predigested samples, and the digestion program was run. After digestion, samples were transferred to 25 mL volumetric flasks which were filled with Type I water from a water purification system (Milli-Q^®^ Integral 3) from Merck (Merck Life Science S.L.U., Madrid, Spain).

The concentrations of Ca, Mg, Na, K, P, Cu, Fe, Mn, and Zn in the sample solutions were analyzed using an ICP-OES SpectroBLUE Twin Interface (SPECTRO Analytical Instruments GmbH, Kleve, Germany). Emission intensities were measured for the most sensitive spectral lines free of interference. Instrumental parameters are detailed in Table 5. Calibration standards for the analysis were prepared from the ICP multi-element standard solution IV Certipur^®^ (Merck KGaA, Darmstadt, Germany). The multi-element reference material used contained certified concentrations for eight metal elements: 1006 ± 20 mg/L Ca, 996 ± 20 mg/L Mg, 1002 ± 20 mg/L Na, 1007 ± 20 mg/L K, 1003 ± 20 mg/L Cu, 1006 ± 20 mg/L Fe, 1008 ± 20 mg/L Mn, and 1006 ± 20 mg/L Zn. The phosphorus reference material utilized was a Plasma CAL/ICP/ICPMS Standard (AnalytiChem Canada Inc., Montreal, QC, Canada) with a certified concentration of 1005 ± 4 mg/L.

The wavelengths selected for the ICP-OES determination of the monitored elements are as follows (λ): Ca 317.933; Mg 279.07; Na 588.995; K 766.491; P 177.491; Cu 327.396; Fe 238.204; Mn 257.611 and Zn 213.856. Results were expressed on a dry weight basis. For the statistical analysis, a one-way ANOVA test was conducted.

## Figures and Tables

**Table 1:** CYN concentrations in potato plants (*S. tuberosum* Phureja Group var Criolla, Colombia) after exposure to four applications of the crude extracts containing CYN by surface or sprinkler irrigation, measured by UPLC-MS/MS. CYN values are expressed as mean ± standard deviation (SD) (*n* = 3).

Irrigation Type	Measured CYN Concentration (µg CYN/g d.w.) Following Irrigation
Control group	ND
Cluster I-Leaf	
Surface irrigation	ND
Sprinkler irrigation	0.13 ± 0.05
Cluster II-Stem	
Surface irrigation	ND
Sprinkler irrigation	0.09 ± 0.04
Cluster III-Tuber	
Surface irrigation	ND
Sprinkler irrigation	ND
Cluster IV-Root	
Surface irrigation	ND
Sprinkler irrigation	ND

dw: dry weight; ND: Not detected.

**Table 2:** Main metabolites found in *S. tuberosum* leaves, stem and tubers, after plant exposure to CYN and determined by UHPLC–MS/MS. The signal colors range from yellow, indicating low intensity, to green, representing high intensity.

Modifications	Composition Change	Error (ppm)	Molecular Weight	RT [min]	Area Max (Splinkler Irrigation)	Area Max (Surface Irrigation)
**Cluster I—leaves**						
Dehydration	-(H_4_O_4_S)	1.45	315.13360	3.141	3.77 × 10^7^	9.80 × 10^7^
Oxidation	-(H_2_O_2_S)	1.55	349.13916	2.662		5.13 × 10^7^
Oxidation, Glycine Conjugation	-(S)+(C_2_ HN)	−0.55	406.15986	2.806		3.99 × 10^7^
Dehydration, Desaturation, Palmitoyl Conjugation	+(C_16_H_26_)	1.45	633.32054	4.616	2.80 × 10^7^	2.64 × 10^7^
Hydration, Nitro Reduction, Sulfation	+(H_4_O_2_S)	−2.65	483.10809	0.839	3.30 × 10^7^	3.30 × 10^7^
Nitro Reduction, Sulfation	+(H_4_O_2_S)	−2.65	483.10809	0.839	3.30 × 10^7^	3.30 × 10^7^
Dehydration, Thiourea to Urea, Glycine Conjugation	-(S)+(C_2_HNO)	−2.98	438.14861	4.336	2.99 × 10^7^	
Reduction, Glucoside Conjugation	-(S)+(C_6_H_10_O_2_)	−1.88	497.21124	4.671		2.14 × 10^7^
Dehydration, Desaturation, Stearyl Conjugation	+(C_18_H_30_)	1.56	661.35195	5.013	7.13 × 10^6^	2.47 × 10^7^
Dehydration	-(H_4_O_4_S)	1.62	315.13365	2.622	1.21 × 10^7^	2.28 × 10^7^
Nitro Reduction, Reduction, Taurine Conjugation	+(C_2_H_9_NS)	1.49	494.16248	2.540	1.53 × 10^7^	1.72 × 10^7^
Nitro Reduction, Thiourea to Urea, Ornitine Conjugation	-(S)+(C_5_H_12_N_2_)	2.07	483.24515	6.866	8.94 × 10^6^	2.12 × 10^7^
Hydration, Reduction	-(O_2_S)+(H_2_)	0.35	353.17004	3.787	2.12 × 10^7^	2.58 × 10^6^
Thiourea to Urea	-(S)+(O)	−2.12	399.13817	2.643		2.00 × 10^7^
Dehydration, Thiourea to Urea, Methylation	-(S)+(C)	−0.23	395.14401	0.922	2.08 × 10^7^	1.91 × 10^7^
Reduction, Acetylation	-(O_2_S)+(C_2_H_2_)	−1.79	377.16924	5.206		2.01 × 10^7^
Reduction	-(O_3_S)	0.53	335.15953	1.319		2.00 × 10^7^
Oxidative Deamination to Ketone, Thiourea to Urea, Arginine Conjugation	-(S)+(C_6_H_9_N_3_O_3_)	1.59	554.20937	3.435		1.86 × 10^7^
Cysteine Conjugation	-(O)+(C_3_H_5_N)	0.14	454.16352	2.160		1.55 × 10^7^
Oxidation, Glucuronide Conjugation	-(S)+(C_6_H_6_O_4_)	−1.02	525.17017	1.427		1.60 × 10^7^
Dehydration, Desaturation, Palmitoyl Conjugation	+(C_16_H_26_)	1.63	633.32065	4.587	1.71 × 10^7^	
Hydration, Glucoside Conjugation	-(S)+(C_6_H_10_O_3_)	−1.70	513.20622	4.295	1.46 × 10^7^	3.17 × 10^6^
Hydration, Taurine Conjugation	+(C_2_H_9_NO_4_S)	1.07	558.14199	2.180	1.33 × 10^7^	1.57 × 10^7^
Hydration, Methylation	-(O_2_S)+(CH_2_)	0.56	365.17012	2.364	1.27 × 10^7^	
Desaturation	-(H_4_O_3_S)	−0.04	331.12804	3.104		1.32 × 10^7^
Hydration, Nitro Reduction	-(O_4_S)+(H_2_)	−1.14	321.17972	4.999		1.36 × 10^7^
Nitro Reduction, Nitro Reduction, Thiourea to Urea	-(O_3_S)+(H_4_)	−0.81	339.19038	6.899	8.80 × 10^6^	1.34 × 10^7^
Oxidative Deamination to Alcohol, Thiourea to Urea, Palmitoyl Conjugation	-(NS)+(C_16_H_29_O_3_)	−2.17	638.35131	4.379	2.94 × 10^6^	1.27 × 10^7^
Methylation	-(O_3_S) +(C)	0.81	347.15963	3.909		1.12 × 10^7^
Nitro Reduction, Thiourea to Urea, Acetylation	-(S)+(C_2_H_4_)	−0.13	411.17534	3.920	1.17 × 10^7^	6.90 × 10^6^
Dehydration, Reduction, Thiourea to Urea	-(S)	−1.03	383.14370	4.159	9.90 × 10^6^	
Dehydration, Dehydration, Thiourea to Urea	-(H_4_OS)	0.56	363.11809	1.115		1.11 × 10^7^
Oxidation, Thiourea to Urea, Glycine Conjugation	-(S)+(C_2_H_3_NO_3_)	1255.72	472.74829	4.520	1.14 × 10^7^	7.83 × 10^6^
Thiourea to Urea, Glycine Conjugation	-(S)+(C_2_H_3_NO_3_)	1255.72	472.74829	4.520	1.14 × 10^7^	7.83 × 10^6^
Desaturation, Oxidation	-(H_4_O_2_S)	−0.04	347.12296	0.749	3.94 × 10^6^	1.02 × 10^7^
Oxidative Deamination to Ketone	-(H_5_NO_2_S)	−0.1	332.11204	3.832		1.03 × 10^7^
**Cluster II—stem**						
Nitro Reduction, Arginine Conjugation	-(O_4_S)+(C_6_H_12_N_4_)	−3.11	459.26921	4.296		1.33 × 10^8^
Dehydration, Reduction, Thiourea to Urea	-(S)	0.07	383.14413	4.473	6.20 × 10^7^	2.16 × 10^7^
Hydration, Nitro Reduction, Sulfation	+(H_4_O_2_S)	−2.45	483.10818	0.896	5.27 × 10^7^	
Nitro Reduction, Sulfation	+(H_4_O_2_S)	−2.45	483.10818	0.896	5.27 × 10^7^	
Reduction, Thiourea to Urea, Glycine Conjugation	-(S)+(C_2_H_5_NO_2_)	−2.56	458.17495	4.289	2.18 × 10^7^	2.65 × 10^6^
Reduction, Acetylation	-(O_2_S)+(C_2_H_2_)	−1.35	377.16941	5.214	2.80 × 10^7^	
Desaturation, Nitro Reduction, Stearyl Conjugation	-(O)+(C_18_H_34_)	−1.86	649.38609	5.038	2.69 × 10^7^	
Dehydration, Nitro Reduction, Palmitoyl Conjugation	-(O_2_)+(C_16_H_30_)	−1.75	605.36003	4.968	1.80 × 10^7^	
Nitro Reduction, Thiourea to Urea, Acetylation	-(S)+(C_2_H_4_)	0.05	411.17542	3.941	1.59 × 10^7^	
Oxidation, Thiourea to Urea, Glycine Conjugation	-(S)+(C_2_H_3_NO_3_)	−2.48	472.15422	4.532	1.24 × 10^7^	4.74 × 10^6^
Thiourea to Urea, Glycine Conjugation	-(S)+(C_2_H_3_NO_3_)	−2.48	472.15422	4.532	1.24 × 10^7^	4.74 × 10^6^
Hydration, Reduction	-(O_2_S)+(H_2_)	0.98	353.17026	3.803	1.18 × 10^7^	
Hydration, Glycine Conjugation	-(OS)+(C_2_H_3_N)	−2.91	408.17454	4.651	1.15 × 10^7^	
**Cluster III—tuber**						
Desaturation, Nitro Reduction, Stearyl Conjugation	-(O)+(C_18_H_34_)	−0.87	649.38674	5.020		3.55 × 10^7^
Dehydration, Nitro Reduction, Thiourea to Urea	-(O_2_S)	0.33	351.15438	4.529	3.50 × 10^7^	3.20 × 10^6^
Hydration	-(O_2_S)	0.33	351.15438	4.529	3.50 × 10^7^	3.20 × 10^6^
Hydration, Methylation	-(O_2_S)+(CH_2_)	0.06	365.16994	4.892	3.38 × 10^7^	2.09 × 10^6^
Dehydration, Nitro Reduction, Palmitoyl Conjugation	-(O_2_)+(C_16_H_30_)	−0.79	605.36061	4.950		3.23 × 10^7^
Dehydration, Nitro Reduction, Thiourea to Urea	-(O_2_S)	1.61	351.15483	3.776		2.73 × 10^7^
Hydration	-(O_2_S)	1.61	351.15483	3.776		2.73 × 10^7^
Oxidative Deamination to Ketone	-(H_5_NO_2_S)	1.82	332.11268	3.798	1.98 × 10^7^	7.61 × 10^6^
Oxidative Deamination to Alcohol	-(H_3_NO_2_S)	1.60	334.12825	3.776		1.69 × 10^7^

**Table 3:** Mineral content in control and CYN-exposed potato tubers by surface or sprinkler irrigation. In addition, data on content of these minerals reported in potato (*S. tuberosum*) cultivars by other authors are summarized.

Minerals	Mineral Content (Measured in This Study) (*S. tuberosum*, Phureja Variety) (mg/kg d.w.)			Mineral Content (Various References)	
	Control	CYN Sprinkler Irrigation	CYN Surface Irrigation	[41,42,43,44,45,46] (mg/kg d.w. *)	[47] (mg/kg f.w. **)
Ca	866–1233 (990 ± 210)	594–1129 (811 ± 281)	1831–2260 (1994 ± 232)	250–830	
K	17,745–17,987 (17,845 ± 126)	19,457–20,731 (20,089 ± 637)	18,938–19,315 (19,131 ± 189)	12,540–33,870	
Mg	1203–1270 (1231 ± 35)	1001–1167 (1086 ± 83)	1290–1338 (1319 ± 26)	185–860	
Na	180–201 (189 ± 11)	134–229 (173 ± 50)	290–349 (319 ± 30)	130–150	
P	4090–4436 (4311 ± 192)	3060–3485 (3302 ± 216)	5498–4483 (4982 ± 508)		
Cu	9.65–10.61 (10.04 ± 0.51)	7.68–8.54 (8.15 ± 0.43)	9.90–11.27 (10.739 ± 0.735)	4.30–31	0.505–2.729
Fe	88.41–97.34 (91.96 ± 4.73)	57.74–60.75 (58.95 ± 1.59)	90.21–103.38 (95.90 ± 6.77)	5.25–340	
Mn	7.58–8.59 (8.171 ± 0.529)	4.91–6.20 (5.71 ± 0.70)	7.43–8.93 (8.21 ± 0.75)	6–16.01	0.022–29.894
Zn	55.70–61.06 (58.46 ± 2.69)	43.30–48.38 (46.57 ± 2.83)	55.00–59.21 (57.76 ± 2.38)	12.6-56.51	

* d.w.: dry weight. ** f.w.: fresh weight.

**Table 4:** Summary of the exposure experiment carried out in *S. tuberosum* plants exposed to CYN by surface or sprinkler irrigation.

Plant	Crop Conditions	Germination	Treatment	Irrigation	CYN Concentration	Application	Sample Processing
*S. tuberosum* Phureja Group var. Criolla Colombia	Greenhouse. Semi-controlled conditions, air temperature 17–20 °C, light: dark cycle of 12:12 h (natural light) and air relative humidity of 55%.	Tubers were planted in plastic bags that contained organic soil and quarzitic sand at a 3:1 ratio.	Control (*n* = 9)	Municipal potable water		4 weeks, 3 times per week	Plants were harvested, stored (−80 °C), and lyophilized.
			Surface irrigation (*n* = 9)	Cyanobacterial crude extract (*C. ovalisporum* strain LEGE X-001)	84.65 µg/L (1st application) 33.80 µg/L (2nd aplication) 3.05 µg/L (3rd–4th applications)		
			Sprinkler irrigation (*n* = 9)				

**Table 5:** ICP OES instrumental parameters to determine mineral content in potatoes.

Plasma Power	1350 W
Plasma gas Coolant Flow	14 L/min
Auxiliary gas flow rate	12 L/min
Nebulizer Flow rate	0.82 L/min
Nebulizer	Cross flow
Nebulizer chamber	Scott
Pump speed	30 rpm
Rinse time	45 s
Sample uptake delay	30 s
Replicates	3

## Data Availability

The original contributions presented in this study are included in this article. Further inquiries can be directed to the corresponding author.

## Abbreviations

The following abbreviations are used in this manuscript:

Ca	Calcium
CYN	Cylindrospermopsin
Cu	Copper
DCM	Dichloromethane
DNA	Deoxyribonucleic Acid
Fe	Iron
ICP-OES	Inductively Coupled Plasma Optical Emission Spectroscopy
K	Potassium
LC-MS	Liquid Chromatography–Mass Spectrometry
Mg	Magnesium
Mn	Manganese
MRM	Multiple Reaction Monitoring
MCs	Microcystins
MC-LR	Microcystin-LR
MeOH	Methanol
Na	Sodium
NOD	Nodularine
P	Phosphorus
RNA	Ribonucleic Acid
TFA	Trifluoroacetic Acid
UHPLC-MS/MS	Ultra-High Performance Liquid Chromatography-MS/MS
WHO	World Health Organization
Zn	Zinc

## References

[1] Adamski M., Wołowski K., Kaminski A., Hindáková A. (2020). Cyanotoxin cylindrospermopsin producers and the catalytic decomposition process: A review. Harmful Algae.

[2] Huisman J., Codd G.A., Paerl H.W., Ibelings B.W., Verspagen J.M.H., Visser P.M. (2018). Cyanobacterial Blooms. Nat. Rev. Microbiol..

[3] Codd G.A., Meriluoto J., Metcalf J.S., Meriluoto J., Spoof L., Codd G.A. (2016). Introduction: Cyanobacteria, Cyanotoxins, Their Human Impact, and Risk Management. Handbook of Cyanobacterial Monitoring and Cyanotoxin Analysis.

[4] Chichova M., Tasinov O., Shkodrova M., Mishonova M., Sazdova I., Ilieva B., Doncheva-Stoimenova D., Kiselova-Kaneva Y., Raikova N., Uzunov B. (2021). New Data on Cylindrospermopsin Toxicity. Toxins.

[5] Vilar M., Ferrão-Filho A. (2022). (Eco)Toxicology of Cyanobacteria and Cyanotoxins: From Environmental Dynamics to Adverse Effects. Toxics.

[6] He Z., Chen Y., Huo D., Gao J., Xu Y., Yang R., Yang Y., Yu G. (2023). Combined methods elucidate the multi-organ toxicity of cylindrospermopsin (CYN) on *Daphnia magna*. Environ. Pollut..

[7] Zhang Y., Duy S.V., Munoz G., Sauvé S. (2022). Phytotoxic effects of microcystins, anatoxin-a and cylindrospermopsin to aquatic plants: A meta-analysis. Sci. Total Environ..

[8] Pichardo S., Cameán A.M., Jos A. (2017). In Vitro Toxicological Assessment of Cylindrospermopsin: A Review. Toxins.

[9] World Health Organization (2020). Cyanobacterial Toxins: Cylindrospermopsins. Background Document for Development of WHO Guidelines for Drinking-Water Quality and Guidelines for Safe Recreational Water Environments. https://iris.who.int/bitstream/handle/10665/338063/WHO-HEP-ECH-WSH-2020.4-eng.pdf.

[10] Terao K., Ohmori S., Igarashi K., Ohtani I., Watanabe M.F., Harada K.I., Ito E., Watanabe M. (1994). Electron Microscopic Studies on Experimental Poisoning in Mice Induced by Cylindrospermopsin Isolated from Blue-Green Alga *Umezakia natans*. Toxicon.

[11] Froscio S.M., Humpage A.R., Burcham P.C., Falconer I.R. (2003). Cylindrospermopsin-Induced Protein Synthesis Inhibition and Its Dissociation from Acute Toxicity in Mouse Hepatocytes. Environ. Toxicol..

[12] Scarlett K.R., Kim S., Lovin L.M., Chatterjee S., Scott J.T., Brooks B.W. (2020). Global Scanning of Cylindrospermopsin: Critical Review and Analysis of Aquatic Occurrence, Bioaccumulation, Toxicity and Health Hazards. Sci. Total Environ..

[13] Poniedziałek B., Rzymski P., Wiktorowicz K. (2012). First report of cylindrospermopsin effect on human peripheral blood lymphocytes proliferation in vitro. Cent. Eur. J. Immunol..

[14] Humpage A.R., Fontaine F., Froscio S., Burcham P., Falconer I.R. (2005). Cylindrospermopsin Genotoxicity and Cytotoxicity: Role of Cytochrome P-450 and Oxidative Stress. J. Toxicol. Environ. Health A.

[15] Corbel S., Mougin C., Bouaïcha N. (2014). Cyanobacterial Toxins: Modes of Actions, Fate in Aquatic and Soil Ecosystems, Phytotoxicity and Bioaccumulation in Agricultural Crops. Chemosphere.

[16] Wörmer L., Cirés S., Carrasco D., Quesada A. (2008). Cylindrospermopsin Is Not Degraded by Co-Occurring Natural Bacterial Communities during a 40-Day Study. Harmful Algae.

[17] Gutiérrez-Praena D., Campos A., Azevedo J., Neves J., Freitas M., Guzmán-Guillén R., Cameán A., Renaut J., Vasconcelos V. (2014). Exposure of *Lycopersicon esculentum* to Microcystin-LR: Effects in the Leaf Proteome and Toxin Translocation from Water to Leaves and Fruits. Toxins.

[18] Machado J., Campos A., Vasconcelos V., Freitas M. (2017). Effects of Microcystin-LR and Cylindrospermopsin on Plant-Soil Systems: A Review of Their Relevance for Agricultural Plant Quality and Public Health. Environ. Res..

[19] Weralupitiya C., Wanigatunge R.P., Gunawardana D., Vithanage M., Magana-Arachchi D. (2022). Cyanotoxins Uptake and Accumulation in Crops: Phytotoxicity and Implications on Human Health. Toxicon.

[20] Prieto A.I., Campos A., Cameán A.M., Vasconcelos V. (2011). Effects on Growth and Oxidative Stress Status of Rice Plants (*Oryza sativa*) Exposed to Two Extracts of Toxin-Producing Cyanobacteria (*Aphanizomenon ovalisporum* and *Microcystis aeruginosa*). Ecotoxicol. Environ. Saf..

[21] M-Hamvas M., Máthé C., Vasas G., Jámbrik K., Papp M., Beyer D., Mészáros I., Borbély G. (2010). Cylindrospermopsin and Microcystin-LR Alter the Growth, Development and Peroxidase Enzyme Activity of White Mustard (*Sinapis alba* L.) Seedlings, a Comparative Analysis. Acta Biol. Hung..

[22] Garda T., Riba M., Vasas G., Beyer D., M-Hamvas M., Hajdu G., Tándor I., Máthé C. (2015). Cytotoxic Effects of Cylindrospermopsin in Mitotic and Non-Mitotic *Vicia faba* Cells. Chemosphere.

[23] Llana-Ruiz-Cabello M., Jos A., Cameán A.M., Oliveira F., Barreiro A., Machado J., Azevedo J., Pinto E., Almeida A., Campos A. (2019). Analysis of the Use of Cylindrospermopsin and/or Microcystin-Contaminated Water in the Growth, Mineral Content, and Contamination of *Spinacia oleracea* and *Lactuca sativa*. Toxins.

[24] McElhiney J., Lawton L.A., Leifert C. (2001). Investigations into the inhibitory effects of microcystins on plant growth and the toxicity of plant tissues following exposure. Toxicon.

[25] Freitas M., Azevedo J., Pinto E., Neves J., Campos A., Vasconcelos V. (2015). Effects of Microcystin-LR, Cylindrospermopsin and a Microcystin-LR/Cylindrospermopsin Mixture on Growth, Oxidative Stress and Mineral Content in Lettuce Plants (*Lactuca sativa* L.). Ecotoxicol. Environ. Saf..

[26] Guzmán-Guillén R., Campos A., Machado J., Freitas M., Azevedo J., Pinto E., Almeida A., Cameán A.M., Vasconcelos V. (2017). Effects of *Chrysosporum (Aphanizomenon) ovalisporum* Extracts Containing Cylindrospermopsin on Growth, Photosynthetic Capacity, and Mineral Content of Carrots (*Daucus carota*). Ecotoxicology.

[27] Kittler K., Schreiner M., Krumbein A., Manzei S., Koch M., Rohn S., Maul R. (2012). Uptake of the Cyanobacterial Toxin Cylindrospermopsin in *Brassica* Vegetables. Food Chem..

[28] Cordeiro-Araújo M.K., Chia M.A., Bittencourt-Oliveira M. (2017). Potential Human Health Risk Assessment of Cylindrospermopsin Accumulation and Depuration in Lettuce and Arugula. Harmful Algae.

[29] Abeysiriwardena N.M., Gascoigne S.J.L., Anandappa A. (2018). Algal Bloom Expansion Increases Cyanotoxin Risk in Food. Yale J. Biol. Med..

[30] Kinnear S. (2010). Cylindrospermopsin: A Decade of Progress on Bioaccumulation Research. Mar. Drugs.

[31] Romero-Oliva C.S., Contardo-Jara V., Block T., Pflugmacher S. (2014). Accumulation of Microcystin Congeners in Different Aquatic Plants and Crops—A Case Study from Lake Amatitlán, Guatemala. Ecotoxicol. Environ. Saf..

[32] Van Hassel W.H.R., Tardy E., Cottyn B., Andjelkovic M., Decombel A., Van Wichelen J., Masquelier J., Rajkovic A. (2025). Irrigation-dependent accumulation of microcystin in different crops under mid-scale greenhouse conditions. J. Agric. Food Res..

[33] Devaux A., Kromann P., Ortiz O. (2014). Potatoes for Sustainable Global Food Security. Potato Res..

[34] FAOSTAT (2013). Food Balances. http://www.fao.org/faostat/en/#data/FBS.

[35] Devaux A., Goffart J.-P., Kromann P., Andrade-Piedra J., Polar V., Hareau G. (2021). The Potato of the Future: Opportunities and Challenges in Sustainable Agri-Food Systems. Potato Res..

[36] Ghislain M., Andrade D., Rodríguez F., Hijmans R.J., Spooner D.M. (2006). Genetic Analysis of the Cultivated Potato *Solanum tuberosum* L. Phureja Group Using RAPDs and Nuclear SSRs. Theor. Appl. Genet..

[37] Ñústez C.E. (2018). Papas Diploides: Un Legado Ancestral para la Agricultura en Colombia. Universidad Nacional de Colombia. http://www.papaunc.com/blog/papas-diploides-un-legado-ancestral-para-la-agricultura-en-colombia.

[38] Priya B.N.V., Saiprasad G.V.S. (2022). “Potato”—Powerhouse for many nutrients. Potato Res..

[39] Abdallah M.F., Van Hassel W.H.R., Andjelkovic M., Wilmotte A., Rajkovic A. (2021). Cyanotoxins and Food Contamination in Developing Countries: Review of Their Types, Toxicity, Analysis, Occurrence and Mitigation Strategies. Toxins.

[40] Diez-Quijada L., Guzmán-Guillén R., Prieto A., Llana-Ruiz-Cabello M., Campos A., Vasconcelos V., Jos A., Cameán A. (2018). New Method for Simultaneous Determination of Microcystins and Cylindrospermopsin in Vegetable Matrices by SPE-UPLC-MS/MS. Toxins.

[41] Peña C., Restrepo-Sánchez L.-P., Kushalappa A., Rodríguez-Molano L.-E., Mosquera T., Narváez-Cuenca C.-E. (2015). Nutritional Contents of Advanced Breeding Clones of *Solanum tuberosum* Group Phureja. LWT-Food Sci. Technol..

[42] Burgos G., Amoros W., Morote M., Stangoulis J., Bonierbale M. (2007). Iron and Zinc Concentration of Native Andean Potato Cultivars from a Human Nutrition Perspective. J. Sci. Food Agric..

[43] André C.M., Ghislain M., Bertin P., Oufir M., Herrera M.R., Hoffmann L., Hausman J.-F., Larondelle Y., Evers D. (2007). Andean Potato Cultivars (*Solanum tuberosum* L.) as a Source of Antioxidant and Mineral Micronutrients. J. Agric. Food Chem..

[44] Orellana E., Bastos M.C., Cuadrado W., Zárate R., Sarapura V., Yallico L., Tabra F., Bao D. (2020). Heavy Metals in Native Potato and Health Risk Assessment in Highland Andean Zones of Junín, Peru. J. Environ. Prot..

[45] Bedoya-Perales N.S., Maus D., Neimaier A., Escobedo-Pacheco E., Pumi G. (2023). Assessment of the Variation of Heavy Metals and Pesticide Residues in Native and Modern Potato (*Solanum tuberosum* L.) Cultivars Grown at Different Altitudes in a Typical Mining Region in Peru. Toxicol. Rep..

[46] Dramicanin A., Andric F., Mutic J., Stankovic V., Momirovic N., Milojkovic-Opsenica D. (2021). Content and Distribution of Major and Trace Elements as a Tool to Assess the Genotypes, Harvesting Time, and Cultivation Systems of Potato. Food Chem..

[47] Srek P., Hejcman M., Kunzová E. (2010). Multivariate analysis of relationship between potato (*Solanum tuberosum* L.) yield, amount of applied elements, their concentrations in tubers and uptake in a long-term fertilizer experiment. Field Crops Res..

[48] Silva P., Vasconcelos V. (2010). Allelopathic effect of *Cylindrospermopsis raciborskii* extracts on the germination and growth of several plant species. Chem. Ecol..

[49] Shahid M., Dumat C., Khalid S., Schreck E., Xiong T., Niazi N.K. (2016). Foliar Heavy Metal Uptake, Toxicity, and Detoxification in Plants: A Comparison of Foliar and Root Metal Uptake. J. Hazard. Mater..

[50] Selim T., Elkefafy S.M., Berndtsson R., Elkiki M., El-kharbotly A.A. (2022). Can Potato Crop on Sandy Soil Be Safely Irrigated with Heavy Metal Polluted Water?. Water.

[51] Satchivi N.M., Stoller E.W., Wax L.M., Briskin D.P. (2000). A Nonlinear Dynamic Simulation Model for Xenobiotic Transport and Whole Plant Allocation Following Foliar Application I. Conceptual Foundation for Model Development. Pestic. Biochem. Phys..

[52] Bukovac M.J., Norris R.F. (1968). Foliar Penetration of Plant Growth Substances with Special Reference to Binding by Cuticular Surfaces of Pear Leaves. Agrochimica.

[53] Pereira A.L., Azevedo J., Vasconcelos V. (2017). Assessment of Uptake and Phytotoxicity of Cyanobacterial Extracts Containing Microcystins or Cylindrospermopsin on Parsley (*Petroselinum crispum* L.) and Coriander (*Coriandrum sativum* L.). Environ. Sci. Pollut. Res..

[54] Zhang Y., Whalen J.K., Sauvé S. (2021). Phytotoxicity and bioconcentration of microcystins in agricultural plants: Meta-analysis and risk assessment. Environ. Pollut..

[55] Zhang Y., Duy S.V., Whalen J.J., Munoz G., Sauve S. (2024). Risk quick sketch: Soil captured most anatoxin-a and microcystin-RR rather than cylindrospermopsin and microcystin-LA/-LY. Stoten.

[56] Chen W., Song L., Gan N., Li L. (2006). Sorption, Degradation and Mobility of Microcystins in Chinese Agriculture Soils: Risk Assessment for Groundwater Protection. Environ. Pollut..

[57] Miller M., Critchley M., Hutson J., Fallowfield H. (2001). The Adsorption of Cyanobacterial Hepatotoxins from Water onto Soil during Batch Experiments. Water Res..

[58] Cao Q., Steinman A.D., Yao L., Xie L. (2018). Effects of Light, Microorganisms, Farming Chemicals, and Water Content on the Degradation of Microcystin-LR in Agricultural Soils. Ecotoxicol. Environ. Saf..

[59] Wu H., Zhou J., Zhang S., Gao Y., Wang C., Cong H., Feng S. (2024). Contributions of the bacterial communities to the microcystin degradation and nutrient transformations during aerobic composting of algal sludge. J. Environ. Manag..

[60] Bouaïcha N., Corbel S., Larramendy M.L., Soloneski S. (2016). Chapter 6. Cyanobacterial Toxins Emerging Contaminants in Soils: A Review of Sources, Fate and Impacts on Ecosystems, Plants and Animal and Human Health. Soil Contamination—Current Consequences and Further Solutions.

[61] Rzymski P., Poniedziałek B. (2015). The Surprising World of Cyanobacteria: Cylindrospermopsin Has a Soil Face. J. Phycol..

[62] Sahu G., Thingujam U., Mohanty S., Dash B., Bhuyan B., Kumari A., Rajput V.D., Mandzhieva S.S., Minkina T., Hullebusch E. (2024). Cyanotoxin Pollution in Water Bodies and Soils Imposes Potential Risks to the Surrounding Flora. Emerging Contaminants.

[63] Corbel S., Mougin C., Nélieu S., Delarue G., Bouaïcha N. (2016). Evaluation of the Transfer and the Accumulation of Microcystins in Tomato (*Solanum lycopersicum* Cultivar MicroTom) Tissues Using a Cyanobacterial Extract Containing Microcystins and the Radiolabeled Microcystin-LR (14C-MC-LR). Sci. Total Environ..

[64] Hinojosa M.G., Cascajosa-Lira A., Prieto A.I., Gutiérrez-Praena D., Vasconcelos V., Jos A., Cameán A.M. (2023). Cytotoxic Effects and Oxidative Stress Produced by a Cyanobacterial Cylindrospermopsin Producer Extract versus a Cylindrospermopsin Non-Producing Extract on the Neuroblastoma SH-SY5Y Cell Line. Toxins.

[65] Banker R., Carmeli S., Werman M., Teltsch B., Porat R., Sukenik A. (2001). Uracil Moiety is Required for Toxicity of the Cyanobacterial Hepatotoxin Cylindrospermopsin. J. Toxicol. Environ. Health A.

[66] White P.J., Broadley M.R. (2009). Biofortification of Crops with Seven Mineral Elements Often Lacking in Human Diets–Iron, Zinc, Copper, Calcium, Magnesium, Selenium and Iodine. New Phytol..

[67] Saha A., Zakir H.M., Quadir Q.F., Sarker N., Biswas P., Mallick S. (2024). Human Health Risks of Trace Metals through the Dietary Intake of Potato Tubers and Exposures of Potato Cultivating Soils: A Case Study of Mymensingh District, Bangladesh. J. Trace Elem. Miner..

[68] Busse J.S., Palta J.P. (2006). Investigating the In Vivo Calcium Transport Path to Developing Potato Tuber Using 45Ca: A New Concept in Potato Tuber Calcium Nutrition. Physiol. Plant..

[69] Subramanian N.K., White P.J., Broadley M.R., Ramsay G. (2011). The Three-Dimensional Distribution of Minerals in Potato Tubers. Ann. Bot..

[70] Sandhu A.K., Sharma A.K., Kaur N., Sidhu S.K., Singh R., Zotarelli L., Morgan K., Christensen C., Sharma L.K. (2024). Evaluate the Phosphorus Application Response in Potatoes under High Phosphorus Soil Test in Florida. Farming Syst..

[71] Lahrouni M., Oufdou K., El Khalloufi F., Baz M., Lafuente A., Dary M., Pajuelo E., Oudra B. (2013). Physiological and biochemical defense reactions of *Vicia faba* L.-*Rhizobium* symbiosis face to chronic exposure to cyanobacterial bloom extract containing microcystins. Environ. Sci. Pollut. Res. Int..

[72] McGregor G.B., Sendall B.C., Niiyama Y., Tuji A., Willis A. (2023). *Chrysosporum ovalisporum* is synonymous with the true-branching cyanobacterium *Umezakia natans* (Nostocales/Aphanizomenonaceae). J. Phychol..

[73] Kotai J. (1972). Instructions for Preparation of Modified Nutrient Solution Z8 for Algae.

[74] Guzmán-Guillén R., Prieto A.I., González A.G., Soria-Díaz M.E., Cameán A.M. (2012). Cylindrospermopsin Determination in Water by LC–MS/MS: Optimization and Validation of the Method and Application to Real Samples. Environ. Toxicol. Chem..

[75] Spooner M., Núñez J., Trujillo G., Herrera M., Guzmán F., Ghislain M. (2007). Extensive Simple Sequence Repeat Genotyping of Potato Landraces Supports a Major Reevaluation of Their Gene Pool Structure and Classification. Proc. Natl. Acad. Sci. USA.

[76] Ñústez-López C., Rodríguez-Molano L. (2020). Papa Criolla (*Solanum tuberosum* Grupo Phureja): Manual de Recomendaciones Técnicas para su Cultivo en el Departamento de Cundinamarca. *Bogotá DC: Corredor Tecnológico Agroindustrial CTA-2*. https://repositorio.unal.edu.co/handle/unal/86779.

[77] Legarda L., García R. (2002). Manual de Riego Agrícola.

[78] Guerrero E., Potosí C., Melgarejo L., Hoyos L., Melgarejo L. (2012). Capítulo 7. Manejo agronomico de Gulupa (*Passiflora edulis Sims*) en el marco de las Buenas Prácticas Agrícolas (BPA). Ecofisiología del Cultivo de la Gulupa (Passiflora edulis Sims).

[79] Rodríguez L., Ñústez C., Estrada N. (2009). Criolla Latina, Criolla Paisa y Criolla Colombia, nuevos cultivares de papa criolla para el departamento de Antioquia (Colombia). Universidad Nacional de Colombia, Facultad de Agronomía, Centro Editorial. Agron. Colomb..

[80] Vargas Prieto A., Fajardo Rodríguez C.L., Romero Rodríguez Y.E., Nieves Forero K.Y. (2019). La asociatividad para articular cadenas productivas en Colombia: El caso de los pequeños productores de papa criolla en Subachoque, Cundinamarca. Coop. Desarro..

[81] Tarantino T.B., Barbosa I.S., Lima D.C., Pereira M.G., Teixeira L.S.G., Korn M.G.A. (2017). Microwave-Assisted Digestion Using Diluted Nitric Acid for Multi-element Determination in Rice by ICP OES and ICP-MS. Food Anal. Methods.

